# Thymoquinone Attenuates Aluminum Chloride-Induced Testicular Injury by Inhibiting NLRP3/Caspase 1/IL-1β Inflammasome Signaling and Polarizing the Macrophages Toward Anti-Inflammatory M2 Phenotype

**DOI:** 10.3390/cells14231906

**Published:** 2025-12-01

**Authors:** Heba M. Elhessy, Basma Adel Khattab, Alaa M. Badawy, Hassan Reda Hassan Elsayed, Mansour A. Alghamdi, Hind Zafrah, Mohammed R. Rabei, Ola A. Habotta, Nermeen H. Lashine

**Affiliations:** 1 Department of Anatomy and Embryology, Faculty of Medicine, Mansoura University, Mansoura 35516, Egypt; heba1.elhessy@mans.edu.eg (H.M.E.); dr_basmakhattab99@mans.edu.eg (B.A.K.); dr_alaabadawy@mans.edu.eg (A.M.B.); nermeenlashine@mans.edu.eg (N.H.L.); 2 Department of Anatomy and Embryology, Faculty of Medicine, New Mansoura University, Mansoura 35516, Egypt; 3 Department of Anatomy, College of Medicine, King Khalid University, Abha 62529, Saudi Arabia; m.alghamdi@kku.edu.sa; 4 Genomics and Personalized Medicine Unit, The Center for Medical and Health Research, King Khalid University, Abha 62529, Saudi Arabia; 5 Department of Physiology, College of Medicine, King Khalid University, Abha 62529, Saudi Arabia; heawad@kku.edu.sa; 6 Department of Basic Health Sciences, College of Applied Medical Sciences, Qassim University, Buraydah 51432, Saudi Arabia; ramirabei@mans.edu.eg; 7 Department of Medical Physiology, Faculty of Medicine, Mansoura University, Mansoura 35516, Egypt; 8 Department of Forensic Medicine and Toxicology, Faculty of Veterinary Medicine, Mansoura University, Mansoura 35516, Egypt; ola_ali@mans.edu.eg; 9 Horus Research Center, Horus University, New Damietta 34518, Egypt

**Keywords:** aluminum, testicular injury, inflammasome, NLRP3, caspase-1, IL-1β, macrophage polarization

## Abstract

**Highlights:**

**What are the main findings?**

- Thymoquinone significantly attenuated AlCl_3_-induced testicular injury by reducing oxidative stress, suppressing NLRP3/caspase-1/IL-1β inflammasome activation, and improving histological architecture.
- Thymoquinone shifted testicular macrophage polarization from a predominantly pro-inflammatory CD68^+^ phenotype toward an anti-inflammatory CD163^+^ phenotype, indicating modulation of the innate immune response.

**What are the implications of the main findings?**

- These findings suggest that thymoquinone protects against aluminum-induced testicular damage primarily through coordinated antioxidant, anti-inflammatory, and immunomodulatory actions targeting the NLRP3 inflammasome pathway.
- Thymoquinone may represent a promising adjuvant or preventive strategy to mitigate male reproductive toxicity associated with environmental or occupational aluminum exposure.

**Abstract:**

In recent decades, the quantity of sperm and the quality of semen have decreased, causing an increased incidence of male infertility. The increased exposure to environmental pollutants and metals, including aluminum, is one of the causes. Aluminum is reported to activate the proinflammatory macrophages and the NOD-like receptor pyrin domain-containing 3 (NLRP3) inflammasome pathway in many organs. Thymoquinone (TQ), the bioactive component of Nigella sativa, possesses immunomodulatory, anti-inflammatory, anti-apoptotic, and antioxidant properties. The purpose of this work is to investigate how thymoquinone affects aluminum chloride (AlCl_3_)-induced testicular damage and to study, for the first time, its role in modifying the inflammasome pathway, specifically NLRP3/caspase-1/IL-1β, and in targeting macrophage polarization. Negative control, TQ control (10 mg/kg/d), AlCl_3_ group (100 mg/kg/d), and AlCl_3_ + TQ group were the rat groups. Serum testosterone, FSH, and LH were measured, along with a seminal analysis, evaluation of testicular oxidative stress markers, and assessment of testicular enzymes LDH, SDH, and ACP. NLRP3, caspase-1, IL-1β, CD68, and CD163 immunohistochemical staining, as well as histological alterations, were evaluated. TQ could lower oxidative stress markers, limit the AlCl_3_-induced activation of the NLRP3/caspase-1/IL-1β inflammasome pathway, and induce macrophage polarization toward an M2 anti-inflammatory phenotype, thereby restoring testicular enzymes, histological structure, semen quality, and hormone levels. Thymoquinone attenuates aluminum chloride-induced testicular injury by inhibiting NLRP3/caspase-1/IL-1β inflammasome signaling and polarizing the macrophages toward an anti-inflammatory M2 phenotype.

## 1. Introduction

The global prevalence of male infertility has increased in recent decades, primarily attributed to a progressive decline in sperm concentration and overall semen quality. One possible explanation is the increasing exposure to metals and environmental pollutants, such as aluminum. Because aluminum is naturally present in soil and many products, humans will unavoidably come into contact with it. According to Klein et al., patients with oligozoospermia exhibited a noticeably higher concentration of aluminum in their semen compared to other patients, and cytological investigation revealed that aluminum was present in spermatozoa [1]. Aluminum was discovered to have a negative link with fertility in a new study that examined the seminal aluminum levels, sperm DNA damage, and semen characteristics among the male partners of couples experiencing infertility that cannot be explained [2]. Rodriguez-Diaz et al. found that greater blastocyst rates in the assisted reproductive treatments were associated with lower levels of semen aluminum, demonstrating the harmful effects of aluminum on reproduction [3].

Administration of aluminum to experimental rats results in decreased sperm count, viability, morphology, and motility [4,5,6], decreased testosterone, LH, and FSH [4,7,8], suppression of the essential regulators of testosterone biosynthesis in testicular Leydig cells CYP11A1, STAR, and HSD3B [9], increased testicular oxidative stress [6,7,10], increased endoplasmic reticulum stress and mitochondrial injury [11], suppressed Nrf-2, HO-1 antioxidant pathway [12,13], increased apoptotic index [8,14], and increased mitophagy [10,15]. Aluminum causes significant testicular histopathological impairment, including reduced epithelial thickness and lower Johnsen scores for spermatogenesis, as well as broken bonds between the germinal epithelial cells [4,8,14]. The ultrastructural examinations revealed electron-dense granules in the lysosomes of Sertoli and Leydig cells, which suggested damage to the mitochondria and endoplasmic reticulum [16].

Exposure to AlCl_3_ activates the innate immunity and increases the pro-inflammatory M1 macrophages while decreasing anti-inflammatory CD163^+^ M2 macrophages. Members of the cell surface pattern recognition receptor (PRR) family, damage-associated molecular pattern (DAMP) sensors, are highly conserved receptors that act as innate immune triggers. An “inflammasome,” a downstream inflammatory pathway, is then activated [17]. The NLRP3 inflammasome is the most widely researched. NLRP3 recruits caspase-1, which, upon activation, triggers IL-1β production [18]. M1 macrophages’ activation, oxidative stress, and apoptosis, particularly when mitochondrial DNA is oxidized, have been shown to play a significant role in activating the NLRP3 inflammasome [19,20]. Aluminum induces inflammatory responses mediated by the activated NLRP3 inflammasome in bone, leading to severe bone loss [21], in the urticaria mouse model [22], and in the Alzheimer’s disease model [23]. Adjuvants containing aluminum have been widely used in the manufacturing of vaccines. Researchers have proposed that aluminum activates the pro-inflammatory signaling pathway NLRP3, through which it can adsorb antigens and modulate the immune response. This discovery has gained widespread acceptance in the field of research [24]. Sertoli cells, which are atypical tolerogenic antigen-presenting cells (APCs), can trigger the inflammasome response [25]. The NLRP3 inflammasome is activated in experimental models of varicocele, as reported in a recent study [26]. This implies that in some instances of infertility, NLRP3 activation may play a role [27]. More information is needed to fully understand how aluminum contributes to the induction of testicular NLRP3.

The health benefits of Nigella sativa (black cumin) have been demonstrated in numerous studies. Additionally, Nigella sativa’s bioactive component, thymoquinone, has anti-inflammatory, antioxidant, anti-apoptotic, and immunomodulatory qualities [28]. Thymoquinone has shown positive effects on the testis in experimental models of testicular toxicity induced by chemotherapeutic agents [29,30,31], and against testicular injury induced by varicocele [32], torsion [33], and ischemia-reperfusion injury [33,34], by attenuating apoptosis. TQ could suppress the NLRP3 inflammasome pathway in hyperlipidemia-induced cardiac damage in mice [35], Alzheimer’s disease [36], and breast cancer [37].

This study investigated whether thymoquinone can attenuate testicular damage caused by aluminum chloride and explores, for the first time, its impact on the inflammasome pathway, specifically the NLRP3/caspase-1/IL-1β pathway, as well as macrophage polarization.

## 2. Materials and Methods

### 2.1. Sample Size Calculation

Following Faul et al., the sample size was determined using G*Power 3.1 [38] based on previous studies [9,39]. According to our hypothesis, the MDA means for the four groups were 0.92, 1.12, 1.88, and 2.5; 0.21, 0.76, 1.36, and 1.82 for testosterone; and 55, 71.7, 90.86, and 123.4 for sperm count. Assuming the MDA, testosterone, and sperm count standard deviations were 0.475, 0.62, and 2.8, respectively, the effect sizes (f) would be 1.3236, 0.9804, and 9.0801, respectively. Under these assumptions, the smallest effect size (0.9804) is considered, and sample sizes of 16, 24, and 8 are used for MDA, testosterone, and sperm count, respectively. At the 5% alpha level, attaining 95% power determines the effect sizes. Six rats per group are recommended as the sample size for the one-way ANOVA with four groups. Using the F-test, 24 reaches 95% power, yielding a *p*-value of 0.050.

### 2.2. Ethical Statement

The design of the study complies with Animals in Research: Reporting In Vivo Experiments (ARRIVE) guidelines and follows the National Institutes of Health’s guide for the care and use of laboratory animals, the U.K. Animals (Scientific Procedures) Act, and was approved by Mansoura University Animal Care and Use Committee (MU-ACUC), Egypt (VM.R.24.10.186), on 27 March 2025.

### 2.3. Experimental Design and Animals

This study employed a real experimental in vivo posttest-only control-group design. In the investigation, 24 adult male Sprague-Dawley rats, weighing 250–300 g and 8–10 weeks old, were used. After a week of acclimation, each rat was housed in a cage with unlimited access to food and water in meticulously controlled, pathogen-free conditions (animals were kept within the specified parameters of humidity, temperature, and light). Male rats were used to assess the effects of AlCl_3_ on the testis. Among the rats, four groups were chosen at random. For four weeks, a negative control group (*n* = 6) received daily intraperitoneal olive oil as a vehicle control; a thymoquinone (TQ) control group (*n* = 6) received 10 mg/kg thymoquinone (Sigma Aldrich, St. Louis, MO, USA) [40], dissolved in olive oil, once daily via intraperitoneal injection; and an aluminum chloride (AlCl_3_) group (*n* = 6) received 100 mg/kg of AlCl_3_ (Sigma Aldrich) daily dissolved in distilled water, administered via gavage needle to induce testicular toxicity [41]. Additionally, the AlCl_3_ + TQ group received AlCl_3_ + TQ in the exact same dosages as groups two and three.

### 2.4. Blood Sampling and Hormonal Assay

The rats were weighed and then anesthetized to allow easy blood extraction from the left ventricle using evacuated tubes. After clotting for an hour at room temperature, the blood was centrifuged at 1600× *g* for 15 min at 4 °C. After two hours of collection, sera were frozen at 80 °C. Using ELISA test kits (Biodiagnostic, Giza, Egypt), the concentrations of testosterone, FSH, and LH were determined at 450 nm, 490–630 nm, and 490–630 nm, respectively [42,43]. The FSH/LH ratio was calculated.

### 2.5. Sacrification and Testis Specimen Collection

At the appointed time for each group, the rats were decapitated. After that, the abdominal cavity was opened to access and remove the testes. We pressed down on both sides of the lower abdomen to make the testicles drop. Using tiny scissors, we then created a little lateral abdominal incision. After that, the revealed testes were carefully removed. [44]. Before weighing with a digital balance, both testes were promptly cleansed with cold normal saline and patted dry with filter paper. The right testis was homogenized in 10 mL of ice-cold phosphate-buffered saline (PBS, 0.01 M, pH 7.4) per gram of tissue using a motor-driven homogenizer (Glas-Col, LLC, Terre Haute, IN, USA). The final concentration was 10% *w*/*v*. Following a centrifugation at 10,000 rpm for 15 min at 4 °C, the supernatants were separated for biochemical examination. For histological analysis, the left testis was prepared for examination.

### 2.6. The Concentrations of Oxidative Stress Indicators in the Homogenate of Testicular Tissue

Diagnostic kits (Biodiagnostic Co., Dokki, Giza, Egypt) were utilized to evaluate biomarkers for oxidative stress in the supernatant of testicular homogenates using a colorimetric assay, as per the manufacturer’s instructions. The testicular levels of malondialdehyde (MDA., No: MD.2529) [45] and level of nitric oxide (NO. No: 2533) [46], catalase (CAT. No: CA2517) [47], and superoxide dismutase (SOD., No: SD2521) [46,48] were measured.

### 2.7. Assessment of the Testicular Enzymes Succinate Dehydrogenase (SDH), Lactate Dehydrogenase (LDH), and Acid Phosphatase (ACP)

A testicular homogenate containing 10 mL was centrifuged at 10,000× *g* for 30 min. The activities of SDH, ACP, and LDH were measured using ELISA Kits (MyBioSource, Inc., San Diego, CA, USA, Catalog Nos.: MBS2023295, MBS046840, and MBS9718969, respectively) following the collection of the tissue homogenate’s supernatant per the manufacturer’s instructions. The ACP-to-LDH ratio was then computed.

### 2.8. Sperm Parameters Analysis

#### 2.8.1. Total Sperm Concentration

To count sperm, a careful massage of the right cauda epididymis was performed to release the epididymal fluid onto a microscope slide. The epididymal fluid was mixed with PBS, then aspirated to the “0.5” mark on a white blood cell pipette. One mL of 40% formaldehyde and 5 g of sodium bicarbonate were dissolved in 100 mL of normal saline to prepare the dilution solution. It was slowly blended after being brought up to the pipette’s “11” point. One drop was placed on each of the two sides of Neubauer’s hemocytometer after the initial one or two drops were discarded. The hemocytometer was left in a humid chamber for 5 min to allow the spermatozoa to settle. Sperm counting was done under a 40× objective lens. The average number of sperm was calculated from five primary squares on either side of the hemocytometer. The sperm concentration in the cauda epididymal fluid sample was calculated using the following formula [49]:

sperm count/mL = (dilution factor) × (number of sperm in five squares) × (0.05 × 10^6^)

#### 2.8.2. Sperm Motility

A microscopic slide contains a 10 μL sample. A 20× magnification binocular light microscope (Olympus CX31, Tokyo, Japan) was used to examine 100 spermatozoa. They were categorized as immotile, type A (motile with progressive movement), and type B (motile without progressive movement). To indicate sperm motility, a proportion of the total sperm was utilized [50].

#### 2.8.3. Sperm Morphology (Percentage of Abnormal Forms)

The percentage of aberrant sperm was determined in the epididymal sperm smear stained with eosin. Ninety-five percent ethyl alcohol was used to dilute and fix a drop of semen, which was then spread out on a glass slide. Eosin was used to stain five air-dried smears on glass slides for each group. Of the 200 spermatozoa observed on each plate, the proportion of abnormal sperm was computed [50] (Supplementary Figure S1).

### 2.9. Histopathology

For histological analysis, a portion of each testis was embedded in paraffin after being preserved in Bouin’s solution for H&E. For immunohistochemistry, another portion of the testis was fixed in formaldehyde 10% because Bouin’s strong fixatives can mask antigens and complicate antigen retrieval during immunohistochemistry. The paraffin sections were then cut into 3 µm thick sections. After H&E staining, the slices were mounted on slides for histopathological evaluation [51]. The Olympus SC100 Colour Microscope (Hachioji-shi, Tokyo, Japan) was used to take photomicrographs of the results.

### 2.10. Johnsen Score for Assessment of Spermatogenesis and Testicular Injury

Using a 10-point scoring system, the Johnsen score is a semi-quantitative assessment of spermatogenesis based on the characteristics of the germinal epithelium. Maximum spermatogenesis activity is indicated by a Johnsen score of 10, whereas total absence of germ cells is indicated by a score of 1 [52].

### 2.11. Immunohistochemical Study

Sections that were 3 μm thick were subjected to immunohistochemistry using the immunoperoxidase method [53,54,55]. To suppress endogenous peroxidase, the testis slices were treated with 0.3% hydrogen peroxide in methanol for 10 min after the tissue sections were submitted for paraffin removal. The testis sections were then heated in 10 mM citrate buffer for 10 min at 95 °C to facilitate antigen recovery. The pieces were then allowed to cool for a whole hour. Testis sections were left at 4 °C overnight with primary rabbit polyclonal antibody for NLRP3, caspase-1, IL-1β, CD163 and monoclonal antibody for CD68 (Bioss; bs-10021R, ServiceBio; GB11383, ServiceBio; GB11113, Cat; ER2001-15, and Genemed; 61-0184 respectively) (1:200, 1:1000, 1:1000, 1:200 and 1:100 dilutions, respectively). A mouse-rabbit polydetector (BSB 0268, Bioscience, Santa Barbara, CA, USA) was then used to hold the slides for 30 min. Hematoxylin was used as a counterstain following four minutes of DAB addition. PBS was used as a reagent control in place of the primary antibody. Following cleaning and drying, the sections were examined under a light microscope [56,57]. Positive staining is indicated by dark brown patches in the nucleus or cytoplasm, set against a blue background. The antigen was cytoplasmic in all. Immunohistochemistry was employed rather than Western blotting because it allows spatial localization of signaling pathway reactivity.

### 2.12. Morphometric Analysis

The immunohistochemical investigation’s morphometric analysis was performed using ImageJ (v1.54p) and Fiji (v1.54p) with a color deconvolution plug-in to separate DAB-positive staining [58,59]. A random non-overlapping field (×400) was used to compute the immunopositive area percentage for caspase-1, NLRP3, IL-1β, CD68, and CD163 in each group. Evaluation of immunopositivity was performed in a random 24 microscopic fields in each group, with two fields per section, and two sections per rat, for each of the six rats per group.

### 2.13. Statistical Analysis of the Study Data

The data was analyzed using SPSS (Version 25.0). The Shapiro–Wilk test was applied to assess the normality. Normally distributed data were presented as mean ± 95% confidence interval (CI) and were compared using one-way ANOVA and post hoc tests. The LSD correction was used when equal variance was anticipated, and the Games-Howell adjustment was used otherwise. After determining the median and range, the ordinal and non-normally distributed scale values were compared using the Kruskal–Wallis H test. Any change with a *p*-value below 0.05 was deemed statistically significant.

## 3. Results

### 3.1. Effect of TQ on AlCl_3_-Induced Changes in Rat and Testicular Weight

**Figure 1:**
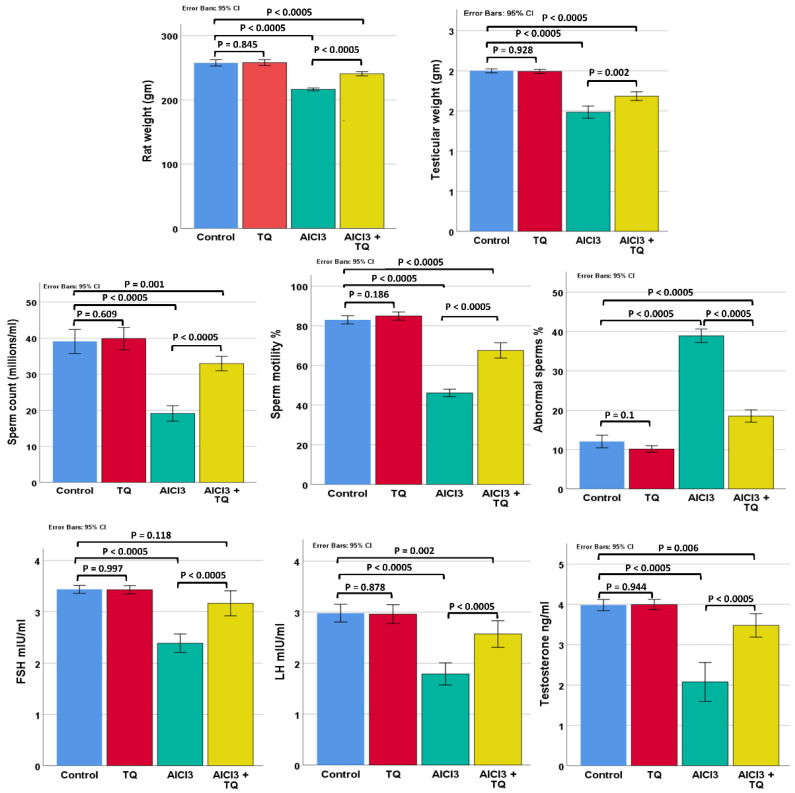
Rat and testicular weights, seminal parameters, and hormonal levels. Results are shown as mean ± 95% confidence interval (CI). ANOVA followed by the LSD test was performed assuming equal variances, except for testicular weight, testosterone, and FSH level, where the Games-Howell test was used. *p* value was <0.0005 for all. F values were 165.508, 166.421, 83.570, 321.313, 507.664, 61.605, 45.118, and 60.928 for rat weight, testicular weight, sperm count, sperm motility, sperm abnormal forms, testosterone, FSH, and LH levels, respectively. Pairwise comparison of significance between the groups is shown in the graph.

The rat and testicular weights showed statistically significant differences (*p* < 0.0005). The negative and TQ groups showed no significant difference. The weights of the AlCl_3_ group were considerably lower than those of the negative control group. Compared to the AlCl_3_ group, the weights of the AlCl_3_ + TQ group were much greater. However, the weights of the AlCl_3_ + TQ groups were still significantly smaller compared to those of the negative control group (Figure 1 and Supplementary Table S1).

### 3.2. Effect of TQ on AlCl_3_-Induced Changes in Epididymal Spermatozoa

The research groups differed significantly in terms of abnormal sperm shapes, motility percentage, and sperm count (*p* < 0.0005). No significant difference was observed between the negative and TQ groups. The number of abnormal sperm forms increased significantly, while the sperm count and proportion of motile sperm decreased substantially in the AlCl_3_ group compared to the negative group. AlCl_3_ + TQ group exhibited a significant decrease in the number of aberrant sperm forms and a substantial rise in the sperm count and the proportion of motile sperm, compared to the AlCl_3_ group. These groundbreaking AlCl_3_ + TQ group values, however, continued to deviate significantly from those of the negative control group (Figure 1 and Supplementary Table S2).

### 3.3. Impact of TQ on the Alterations in Testosterone, FSH, and LH Hormone Levels Brought on by AlCl_3_

Serum testosterone, LH, and FSH levels differed considerably between research groups (*p* < 0.0005). No significant difference was observed between the negative and TQ groups. When hormone levels were investigated in the AlCl_3_ group compared to the negative control group, a substantial decline was found. Hormone levels in the AlCl_3_ + TQ group increased dramatically compared with those in the AlCl_3_ group. The hormone levels of the AlCl_3_ + TQ groups remained much lower than those of the negative group, except for FSH levels, which did not change appreciably (Figure 1 and Supplementary Table S3).

### 3.4. TQ’s Impact on the Alterations in the Testicular Enzymes ACP, LDH, and SDH Induced by AlCl_3_

**Figure 2:**
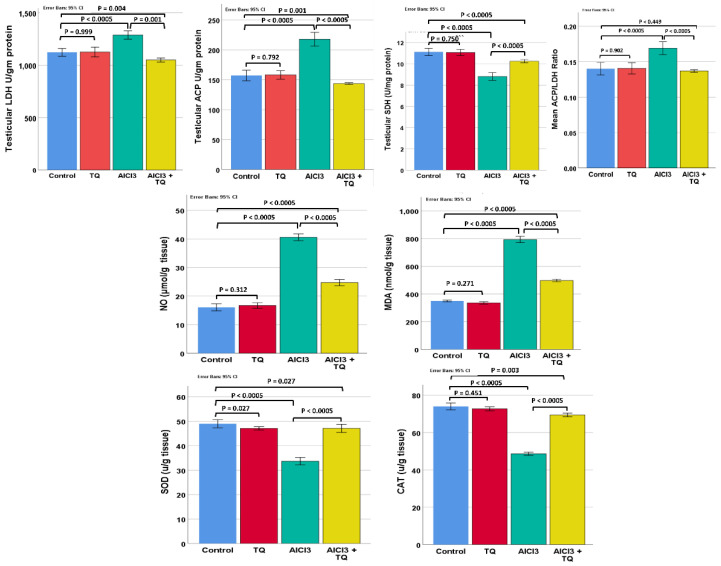
Testicular enzymes and oxidative stress markers. Results are shown as mean ± 95% confidence interval (CI). ANOVA followed by the LSD test was performed assuming equal variances, except for testicular LDH, MDA, and CAT levels, where the Games-Howell test was used. *p* value was <0.0005 for all. F values were 49.091, 110.469, 84.899, 26.281, 684.381, 1532.202, 160.935, and 606.952, for LDH, ACP, SDH, ACP/LDH ratio, NO, MDA, SOD, and CAT, respectively. Pairwise comparison of significance between the groups is shown in the graph.

The research groups showed significant differences in testicular ACP, LDH, SDH, and ACP/LDH ratio (*p* < 0.0005). No significant difference was observed between the negative and TQ groups. The AlCl_3_ group had significantly lower SDH and higher ACP, LDH, and ACP/LDH ratios than the negative group. The AlCl_3_ + TQ group had substantially lower ACP, LDH, and ACP/LDH ratios, as well as a considerably higher SDH, compared to the AlCl_3_ group. However, the enzyme levels in the AlCl_3_ +TQ groups were markedly different from those in the negative control group, except for the ACP/LDH ratio, which showed no significant change (Figure 2 and Supplementary Table S4).

### 3.5. TQ’s Impact on AlCl_3_-Induced Changes in Testicular Oxidative Markers: CAT, MDA, SOD, and NO

Testicular CAT, MDA, SOD, and NO exhibited significant differences (*p* < 0.0005). An insignificant difference was found between the negative and TQ groups. The AlCl_3_ group showed a significant decrease in testicular SOD and CAT and a substantial increase in NO and MDA compared with the negative control group. The AlCl_3_ + TQ group exhibited significantly higher levels of CAT and SOD and lower levels of NO and MDA, compared to the AlCl_3_ group. However, these markers in the AlCl_3_ + TQ groups exhibited substantial changes compared to the negative control group (Figure 2 and Supplementary Table S5).

### 3.6. TQ’s Impact on Testicular Histological Alterations Brought on by AlCl_3_, Observed by H&E Staining

**Figure 3:**
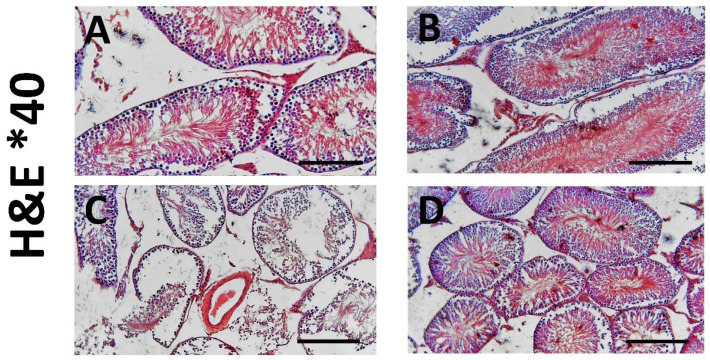
(**A**–**D**) Testicular histopathology as shown by hematoxylin and eosin (×40) of the negative control, TQ, AlCl_3_, and AlCl_3_ + TQ groups ((**A**), (**B**), (**C**), and (**D**), respectively). Scale bar = 200 µm. H&E of the negative control and TQ groups showed standard testicular tubular and interstitial architecture. The AlCl_3_ group showed tubular atrophy, cellular sloughing, and separation. The AlCl_3_ + TQ group showed relative restoration of the standard testicular architecture. The chart shows the results among the study groups.

**Figure 4:**
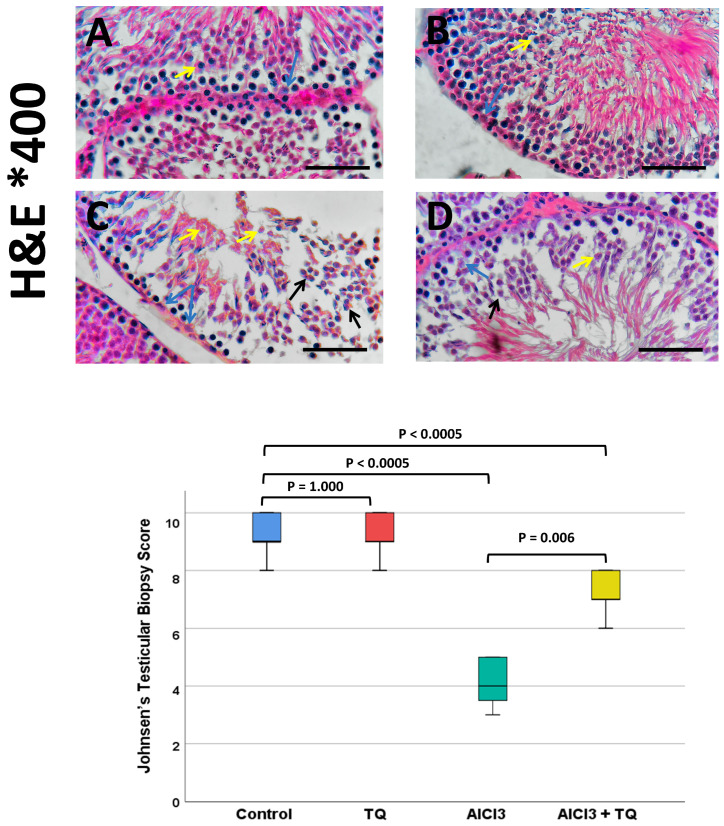
(**A**–**D**) Testicular histopathology as shown by hematoxylin and eosin (×400) of the negative control, TQ, AlCl_3_, and AlCl_3_ + TQ groups ((**A**), (**B**), (**C**), and (**D**), respectively). Scale bar = 50 µm. H&E of the negative control and TQ groups showed standard testicular tubular and interstitial architecture. The AlCl_3_ group showed apoptotic germ cells, separated Sertoli cells, cellular sloughing, inflammatory infiltrate, and edema. The AlCl_3_ + TQ group showed relative restoration of the standard testicular architecture. Germ cells (yellow arrows), Sertoli cells (blue arrows), cellular sloughing (black arrows). The chart shows the results among the study groups. A statistically significant difference in the Johnsen score was demonstrated among the study groups using the Kruskal–Wallis test (*p* < 0.0005, H = 77.990). Pairwise comparisons revealed no significant difference between the negative control and TQ groups. The Johnsen score was significantly lower in the AlCl_3_ group than in the negative control group. However, the AlCl_3_ + TQ group showed a significant increase in the Johnsen score compared to the AlCl_3_ group.

The negative control and TQ groups showed standard testicular tubular and interstitial architecture. The AlCl_3_ group demonstrated tubular atrophy, cellular sloughing, germ cell degeneration, apoptotic spermatogonia, loss of primary spermatocytes, inflammatory cell infiltrate, edema, and Sertoli cell separation. The testicular architecture was restored in the AlCl_3_ +TQ group (Figure 3 and Figure 4).

### 3.7. TQ’s Impact on AlCl_3_-Induced Changes in the Johnsen Score for Spermatogenesis

The Johnsen score differed significantly across the groups (*p* < 0.0005). A pairwise analysis of the groups using the Kruskal–Wallis test showed no significant difference between the negative and TQ groups. The AlCl_3_ group had a significantly lower Johnsen score than the negative control group. However, the AlCl_3_ + TQ group demonstrated a significant increase in the Johnsen score compared to the AlCl_3_ group (Figure 4).

### 3.8. Impact of TQ on AlCl_3_-Induced Alterations in NLRP3, Capase-1, IL-1β, CD68, and CD163 Immunohistochemistry Expression

**Figure 5:**
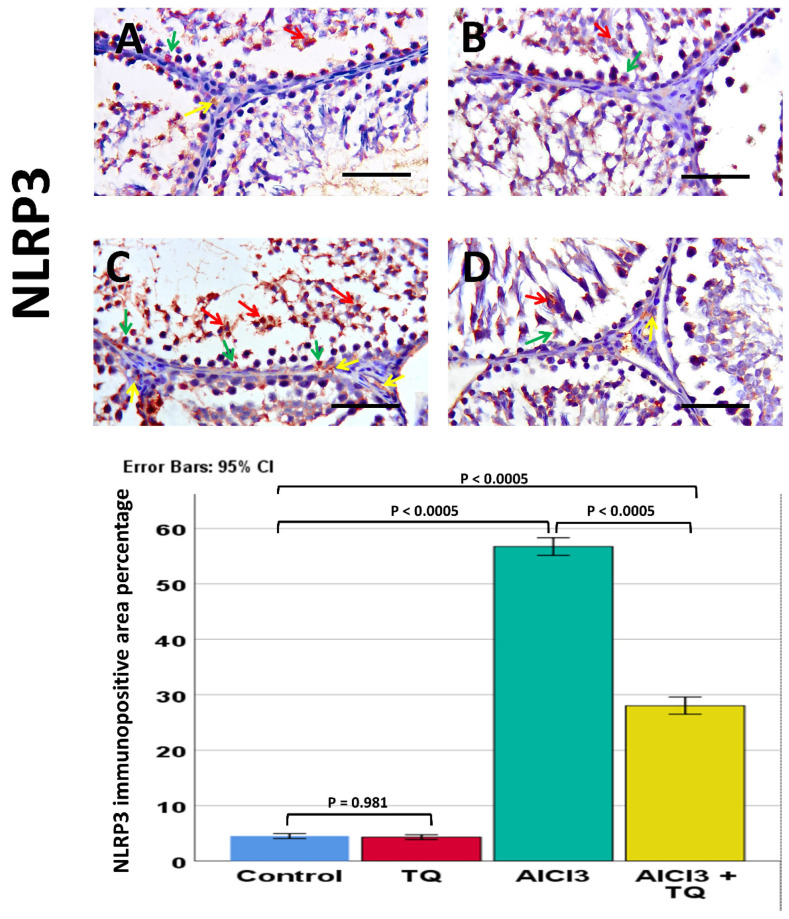
The TQ effect on testicular NLRP3 immunohistochemical expression in AlCl_3_-treated rats (×400) of the negative control, TQ, AlCl_3_, and AlCl_3_ + TQ groups ((**A**), (**B**), (**C**), and (**D**), respectively). Scale bar = 50 µm. The figure shows mild cytoplasmic expression of NLRP3 in germ cells, Sertoli, and Leydig cells in the negative control and TQ groups, intense expression in the AlCl_3_ group, and moderate expression in the AlCl_3_ + TQ group. Red arrows: germ cells, green arrows: Sertoli, and yellow arrows: Leydig cells. The histogram displays the effect of TQ on the percentage of NLRP3-positive areas. The results are shown as mean ± 95% confidence interval (CI). ANOVA followed by the Games-Howell test was used as equal variance was not assumed. *p* value was <0.0005. The F value was 457.403. Pairwise comparison of significance between the groups is shown in the graph.

**Figure 6:**
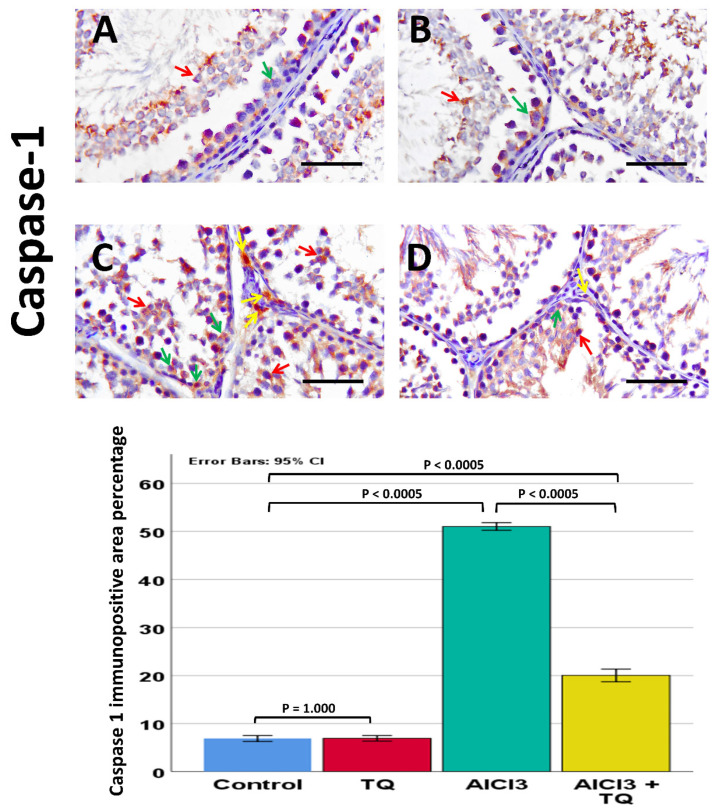
The TQ effect on caspase-1 immunohistochemical expression in the testis of AlCl_3_-treated rats (×400) of the negative control, TQ, AlCl_3_, and AlCl_3_ + TQ groups ((**A**), (**B**), (**C**), and (**D**), respectively). Scale bar = 50 µm. The figure shows a mild expression of caspase-1 in the negative control and TQ groups, an intense cytoplasmic expression in germ cells, Leydig cells, and Sertoli cells of the AlCl_3_ group, and a moderate expression in the AlCl_3_ + TQ group. Red arrows: germ cells, green arrows: Sertoli, and yellow arrows: Leydig cells. The histogram displays the effect of TQ on the percentage of caspase-1-positive cells. The results are noted as mean ± 95% confidence interval (CI). ANOVA followed by the Games-Howell test was used as equal variance was not assumed. *p* value was <0.0005. F value was 561624. Pairwise comparison of significance between the groups is shown in the graph.

**Figure 7:**
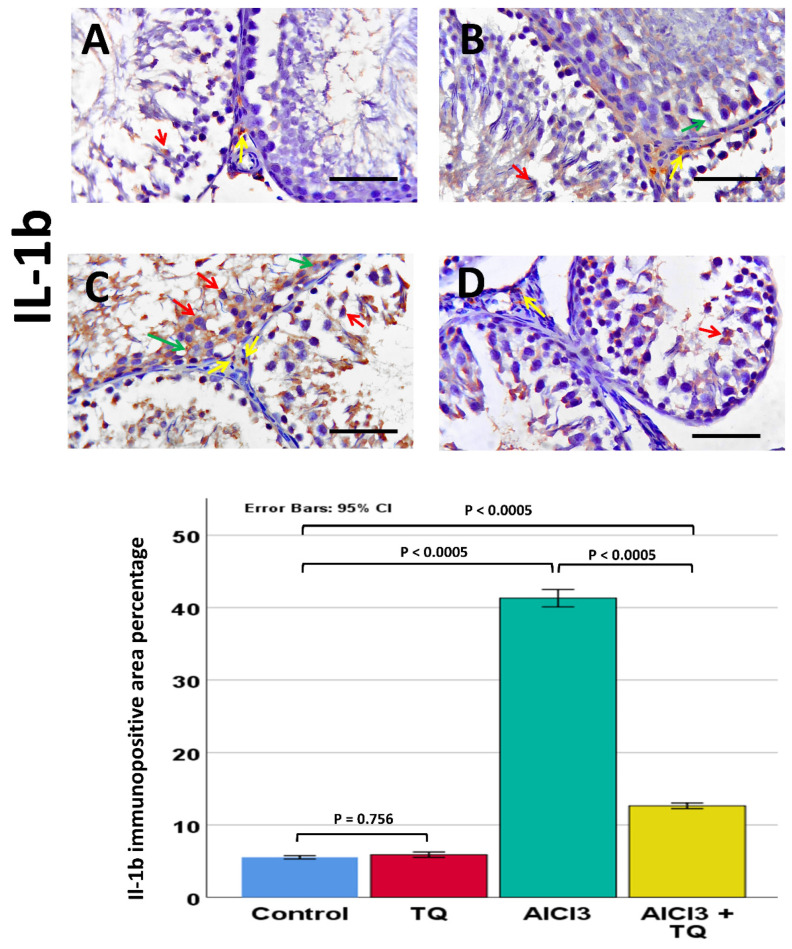
The TQ effect on IL-1β immunohistochemical expression in the testis of AlCl_3_-treated rats (×400) of the negative control, TQ, AlCl_3_, and AlCl_3_ + TQ groups ((**A**), (**B**), (**C**), and (**D**), respectively). Scale bar = 50 µm. The figure shows mild cytoplasmic IL-1β expression in Leydig, Sertoli, and germ cells in the negative control and TQ groups, intense expression in the AlCl_3_ group, and moderate expression in the AlCl_3_ + TQ group. Red arrows: germ cells, green arrows: Sertoli, and yellow arrows: Leydig cells. The histogram shows the effect of TQ on the percentage of IL-1β-positive area. The results are mentioned as mean ± 95% confidence interval (CI). ANOVA followed by the Games-Howell test was used as equal variance was not assumed. *p* value was <0.0005. The F value was 647.413. Pairwise comparison of significance between the groups is shown in the graph.

**Figure 8:**
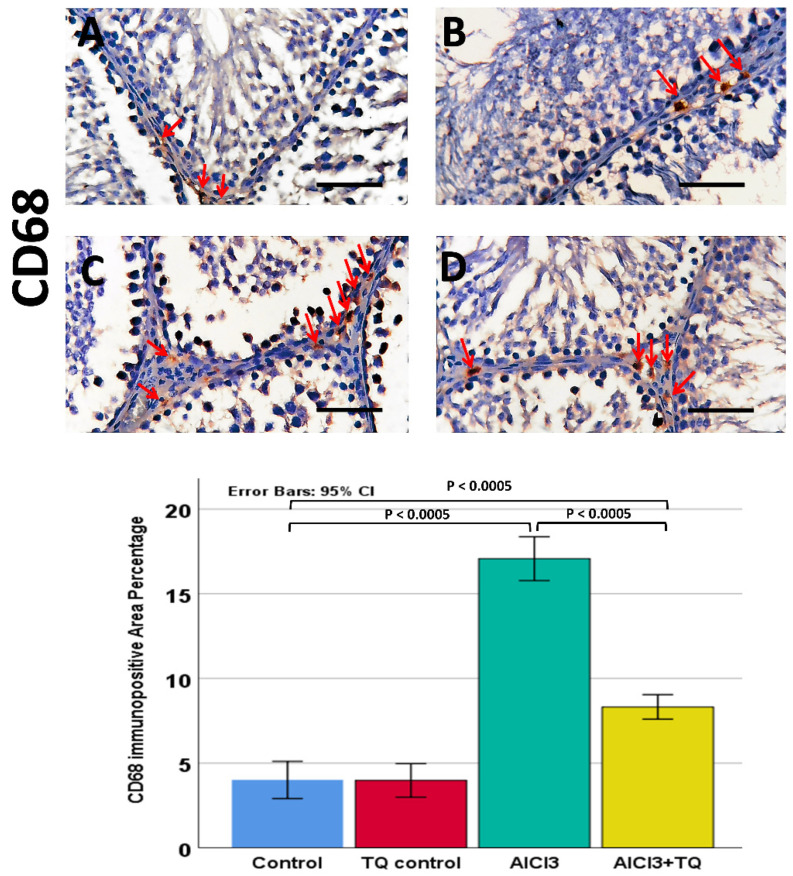
The TQ effect on CD68 immunohistochemical expression in the testis of AlCl_3_-treated rats (×400) of the negative control, TQ, AlCl_3_, and AlCl_3_ + TQ groups ((**A**), (**B**), (**C**), and (**D**), respectively). Scale bar = 50 µm. The figure shows mild cytoplasmic CD68 expression in macrophages in the interstitial tissue of the negative control and TQ groups, intense expression in the AlCl_3_ group, and moderate expression in the AlCl_3_ + TQ group. Red arrows: CD68+ macrophages. The histogram shows the effect of TQ on the percentage of CD68+ve area. The results are noted as mean ± 95% confidence interval (CI). ANOVA was followed by the Games-Howell test, as equal variance was not assumed. *p* value was <0.0005. The F value was 228.829. Pairwise comparison of significance between the groups is shown in the graph.

**Figure 9:**
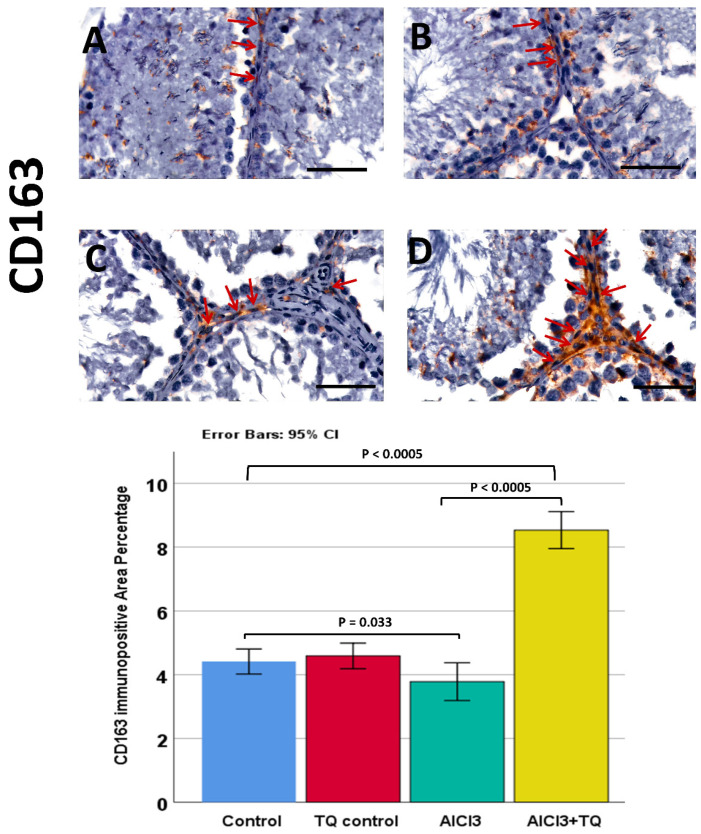
The TQ effect on CD163 immunohistochemical expression in the testis of AlCl_3_-treated rats (×400) of the negative control, TQ, AlCl_3_, and AlCl_3_ + TQ groups ((**A**), (**B**), (**C**), and (**D**), respectively). Scale bar = 50 µm. The figure shows mild cytoplasmic CD163 expression in macrophages of the interstitial tissue in the negative control, TQ, and AlCl_3_ groups, and intense expression in the AlCl_3_ + TQ group. Red arrows: CD163+ macrophages. The histogram shows the effect of TQ on the percentage of CD163+ve area. The results are noted as mean ± 95% confidence interval (CI). ANOVA was followed by the Games-Howell test, as equal variance was not assumed. *p* value was <0.0005. The F value was 122.877. Pairwise comparison of significance between the groups is shown in the graph.

Caspase-1 was observed in germinal cells, Leydig, and endothelial cells. NLRP3 was detected in germ cells, Leydig cells, and Sertoli cells, whereas IL-1β was present in all three cell types. Notably, NLRP3, IL-1β, and caspase-1 exhibited high expression in the AlCl_3_ group. On the other hand, the AlCl_3_ + TQ group had considerable immunoreactivity (Figure 5, Figure 6 and Figure 7). CD68 showed mild expression in the macrophages of negative and TQ controls, intense expression in the AlCl_3_ group, and moderate expression in the AlCl_3_ + TQ group (Figure 8). CD163 showed mild expression in M2 macrophages of negative, TQ controls, and AlCl_3_ group, and intense expression in the AlCl_3_ + TQ group (Figure 9).

### 3.9. Morphometric Analysis of the Immunohistochemical Expression of NLRP3, IL-1β, Caspase-1, CD68, and CD163

Testicular NLRP3, IL-1β, caspase-1, CD68, and CD163 immunohistochemical reactivity varied significantly (*p* < 0.0005) between the study groups. There was an insignificant difference between the negative and TQ control groups. The AlCl_3_ group showed significantly higher expression of NLRP3, IL-1β, caspase-1, and CD68, and significantly lower CD163 expression than the negative control group. The AlCl_3_ + TQ group exhibited a significant reduction in NLRP3, IL-1β, caspase-1, and CD68, and a significant elevation in the expression of CD163 compared to the AlCl_3_ group (Figure 5, Figure 6, Figure 7, Figure 8 and Figure 9 and Supplementary Table S6).

## 4. Discussion

Male infertility has increased over recent decades, with deteriorating semen quality. One of the main factors is the inevitable rise in exposure to environmental elements, such as aluminum. We examined the impact of AlCl_3_ on testis and semen parameters. The dose of AlCl_3_ used in our study (100 mg/kg/day) was markedly higher than typical human dietary or environmental exposure levels. In 2011, the Joint FAO/WHO Expert Committee on Food Additives (JECFA) established a provisional tolerable weekly intake (PTWI) for aluminum as 2 mg/kg bw [60]. However, our high-dose model was designed to simulate high occupational exposure rather than routine human exposure. Workers in aluminum smelting, welding, and pigment industries, as well as those handling aluminum salts such as AlCl_3_ in water treatment and ceramics manufacturing, are known to have significantly higher systemic aluminum levels than the general population. [61]. Many previous studies have used these higher doses to mimic high-exposure toxicity, enabling more precise observation of aluminum-induced oxidative and histopathological changes. [62,63,64]. In our study, AlCl_3_ was administered orally, which closely mimics the most common route of human exposure to aluminum, including dietary intake, drinking water, and food additives.

In line with earlier results, a significant increase in the number of aberrant sperm forms and a substantial decrease in the sperm count and percentage of motile sperm were found in the AlCl_3_ group [5,6]. Patients with oligozoospermia exhibited significantly greater aluminum concentrations [1]. Seminal aluminum level is correlated with sperm DNA damage, impaired semen quality, and unexplained infertility [2]. Inflammation in the testis and peroxidation of sperm membrane polyunsaturated fatty acids, which are critical for sperm viability, may be the leading causes of the reported inhibition of spermatogenesis, sperm impairments, and histological changes [65].

In line with earlier findings, administering aluminum resulted in a statistically significant drop in the rat body and testicular weights [4,66]. This reduction indicates a combination of systemic toxicity and testicular damage. The testicular weight loss was likely due to oxidative injury, germ cell depletion, and Leydig cell dysfunction. However, some other studies reported insignificant changes in body and testicular weights [67], maybe due to variations in exposure time, dosage, and assay methods.

According to earlier reports, aluminum significantly impaired the structure of the testicles, causing vacuolization of germinal epithelium, congestion, interstitial edema, decreased epithelial thickness, and Johnsen scores, broken bonds of the germinal cells, Leydig cell degeneration, and the detachment and degeneration of immature germ cells [4,8,14]. In addition, embryonic exposure to aluminum disrupts testicular development by disrupting the cytoskeletal protein homeostasis [68].

The testes and spermatozoa are vulnerable to oxidative stress due to the high concentration of polyunsaturated fatty acids and their limited antioxidant capacity. Aluminum administration increased testicular oxidative stress, as evidenced by elevated testicular NO levels, MDA levels, and decreased SOD and CAT levels, consistent with previous findings [6,10]. Aluminum was reported to cause endoplasmic reticulum stress and mitochondrial injury [11], and suppress Nrf-2 and the HO-1 antioxidant pathway [12,13].

AlCl_3_-induced testicular toxicity disrupts cellular metabolism and damages spermatogenic cells, as evidenced by altered enzyme activities in our study. The increase in testicular LDH, a cytoplasmic enzyme released during cell membrane damage or anaerobic metabolism, indicates cellular injury and hypoxia within the testicular tissue, following previous reports [69]. The elevated testicular acid phosphatase (ACP) suggests lysosomal membrane destabilization, which is linked to germ cell degeneration and autolytic processes [69]. In contrast, decreased SDH, a mitochondrial enzyme involved in the Krebs cycle, reflects mitochondrial dysfunction and impaired oxidative metabolism, thereby reducing ATP production—a critical factor for spermatogenesis. Together, these enzyme changes signal testicular cell damage, metabolic stress, and compromised fertility potential in testes exposed to aluminum. However, other studies showed a decrease in testicular LDH and ACP [70]. The variability in LDH and ACP levels reported across studies may reflect differences in the stage of testicular injury, with early damage leading to enzyme release and elevation. At the same time, advanced atrophy leads to cell depletion and reduced enzyme activity.

Additionally, variations in dosage, exposure time, and assay methods likely contribute to these discrepancies. The ACP/LDH ratio showed a statistically significant increase with aluminum administration, suggesting increased lysosomal involvement relative to cytoplasmic leakage, consistent with chronic damage where autophagic/lysosomal pathways are activated. It also reflects persistent stress, inflammation, or degenerative changes involving Sertoli and germ cells.

Administration of aluminum to experimental rats resulted in reduced levels of testosterone, LH, and FSH, as previously reported [4,7,8], by disrupting the hypothalamic-pituitary–gonadal axis, which impairs Leydig and Sertoli cell function, testosterone production, and spermatogenesis, as evidenced by histopathological changes such as seminiferous tubule degeneration, Leydig cell atrophy, and germ cell apoptosis. The hormonal decline translates into functional reproductive deficits, including reduced sperm count, motility, and testicular weight, ultimately contributing to testicular atrophy and infertility. Additionally, aluminum suppresses the essential regulators of testosterone biosynthesis in testicular Leydig cells, including CYP11A1, STAR, and HSD3B [9]. However, other studies reported an increase in FSH as a negative feedback mechanism for the decreased testosterone [71], while others reported insignificant changes in hormonal levels. Therefore, the route of administration, optimal dosage, and duration required to establish the model need further elaboration [67,72].

Aluminum exposure triggers innate immune responses, leading to macrophage activation. The M1 proinflammatory macrophage (CD68+) phenotype greatly increases after AlCl_3_ intake and plays a vital role in testicular inflammation. This increase activates inflammasomes and releases inflammatory mediators, such as TNF-α and IL-6, thereby disrupting the blood–testis barrier (BTB), promoting lymphocyte infiltration, and causing germ cell death. Impaired spermatogenesis and Leydig cell function result in diminished steroidogenesis and testosterone release, thereby exacerbating testicular damage [73]. CD163+ anti-inflammatory M2 macrophages infiltrate the testis during toxic injuries, aiming to regulate inflammation and facilitate tissue healing. However, in cases of severe or ongoing injury caused by AlCl_3_, macrophage reparative efforts are overwhelmed by persistent inflammation, limiting their ability to restore tissue homeostasis. As a result, despite their purpose to lower inflammation, M2-like macrophages are rendered ineffective by the dominance of pro-inflammatory signals, resulting in long-term testicular injury [74]. Therefore, upon exposure to AlCl_3_, the balance shifts toward CD68+ (M1-like) pro-inflammatory macrophages, which activate the inflammasome, disrupt spermatogenesis, and affect Leydig cell function. Although CD163+ (M2-like) macrophages are present, they are insufficient to alleviate the harm generated in this milieu entirely [73,74].

Members of the cell surface pattern recognition receptor (PRR) family, damage-associated molecular pattern (DAMP) sensors, are highly conserved receptors that act as innate immune triggers. An “inflammasome,” a downstream inflammatory pathway, is then activated [17]. The NLRP3 inflammasome is the most widely researched. NLRP3 recruits caspase-1 which, upon activation, triggers the production of IL-1β [18]. Oxidative stress and apoptosis, particularly when mitochondrial DNA is oxidized, have been shown to play a significant role in activating the NLRP3 inflammasome [19,20]. Aluminum has been reported to trigger inflammatory responses, which are mediated by NLRP3 inflammasome activation in bone, leading to severe bone loss [21], in the urticaria mouse model [22], and a model of Alzheimer’s diseases in the brain [23]. Adjuvants containing aluminum are frequently used in the manufacturing of vaccines. According to the researchers, aluminum may absorb antigens by triggering the pro-inflammatory signaling pathway NLRP3, which modifies immune system reaction and antigen stability [24].

Sertoli cells, non-professional tolerogenic APCs, may also initiate inflammation via the inflammasome pathway, NLRP3/caspase-1/IL-1ß, meaning that common APCs, e.g., macrophages, are not the only ones capable of doing so [25]. This suggests that certain infertility cases may be caused by an activated NLRP3 inflammasome [26,27]. In our research, we demonstrate for the first time that AlCl_3_ administration induces the testicular NLRP3/caspase-1/IL-1β pathway, as observed by immunohistochemical staining. Caspase-1 was highly reactive in germ cells, Leydig cells, and endothelial cells. NLRP3 was highly reactive in germ cells, Leydig cells, and Sertoli cells. IL-1β was highly reactive in germ cells, Leydig cells, and Sertoli cells. IL-1β is initially produced as an inactive precursor (pro-IL-1β), which is subsequently cleaved by activated caspase-1 to generate the mature, biologically active IL-1β. The simultaneous upregulation of NLRP3 and caspase-1 immunopositivity in the AlCl_3_-treated group supports the activation of the inflammasome pathway responsible for the conversion of pro-IL-1β to mature IL-1β.

The benefits of black cumin (Nigella sativa) have been demonstrated in several studies. Additionally, Nigella sativa’s bioactive component, thymoquinone (TQ), exhibits antioxidant, anti-inflammatory, anti-apoptotic, and immunomodulatory properties [28]. The efficacy of TQ in reducing testicular damage caused by AlCl_3_ was examined in this study. Our objective was not to establish a full therapeutic dose–response curve, but rather to determine whether thymoquinone (TQ) at a previously established effective dose could mitigate the testicular injury induced by AlCl_3_. The selected dose of 10 mg/kg/day was chosen based on multiple earlier studies demonstrating consistent antioxidant, anti-inflammatory, and reproductive-protective effects of TQ at this level in rodent models of oxidative or toxic injury [75,76,77]. Doses in this range (5–10 mg/kg/day) are widely regarded as biologically active yet non-toxic, and thus suitable for assessing mechanistic endpoints without confounding systemic effects. In comparison, higher doses (>20 mg/kg/day) did not produce proportionally greater protection and, in some reports, increased systemic stress.

Thymoquinone was able to mitigate several testicular histopathological changes induced by AlCl_3_, including cellular desquamation, tubular atrophy, germ cell degeneration, Leydig cell hyperplasia, detachment of Sertoli cells, and apoptosis of spermatogonia and primary spermatocytes, while also improving the Johnsen’s score. Increased sperm motility and count, and a decrease in aberrant sperm forms, indicate that semen quality also improves. As evidenced by enhanced CAT and SOD levels and decreased testicular NO and MDA levels, TQ could reduce testicular oxidative stress. TQ raised FSH, LH, and testosterone levels, while lowering testicular enzymes ACP and LDH and increasing SDH. TQ’s protective effect against testicular injury caused by AlCl_3_ is comparable to that documented against testicular injury caused by chemotherapeutic drugs, such as cisplatin [30,31], methotrexate [29,78], cyclophosphamide [40], bleomycin [79], and against testicular injury induced by varicocele [32], torsion [33], and ischemia-reperfusion injury [34].

In the current study, thymoquinone was found to suppress the testicular NLRP3/caspase-1/IL-1β pathway, as demonstrated by immunohistochemistry. In line with earlier findings in various experimental models, TQ reduced NLRP3 expression in Sertoli and Leydig cells, as well as caspase-1 in Leydig, macrophage, fibroblast, and endothelial cells, and IL-1β in Leydig, Sertoli, and germ cells. TQ could suppress the NLRP3 inflammasome pathway in the hyperlipidemia-induced cardiac damage in mice [35], Alzheimer’s disease [36], and breast cancer [37]. Furthermore, TQ may inhibit NLRP3 and pyroptosis while enhancing the survival of perforator skin flaps [80].

The mechanisms by which thymoquinone suppresses the activation of the NLRP3 inflammasome and its downstream signaling (caspase-1, IL-1β, and IL-18) may include maintaining mitochondrial integrity and inhibiting oxidative stress, as thymoquinone reduces reactive oxygen species (ROS) generation, since ROS are crucial activators of the NLRP3 inflammasome. Another mechanism is the TQ role in modulating NF-κB signaling: thymoquinone suppresses NF-κB activation, which is responsible for the transcriptional priming of NLRP3 and pro-IL-1β. Moreover, TQ has a direct anti-inflammatory effect as it can inhibit caspase-1 enzymatic activity and reduce IL-1β maturation in several models of tissue injury and inflammation [81]. TQ could alter macrophage responses and reduce pro-inflammatory CD68+ macrophage accumulation while maintaining the balance of anti-inflammatory CD163+ macrophages, thereby facilitating tissue healing and minimizing inflammation-related damage in the testis [82]. TQ concurrently modulates oxidative stress markers, inflammasome activation, and macrophage polarization, suggesting an interrelated antioxidant and anti-inflammatory action. The exact sequence of events and primary molecular targets of TQ remain to be elucidated.

Thymoquinone could help restore testicular enzyme levels, including LDH, which indicates membrane stabilization and less cell damage; SDH, which suggests optimized mitochondrial activity and energy metabolism in germ and Sertoli cells; ACP, which means less autolysis, lysosomal stability, and cell degradation; and the ACP/LDH ratio, which indicates testicular damage resolution [83].

## 5. Conclusions

**Figure 10:**
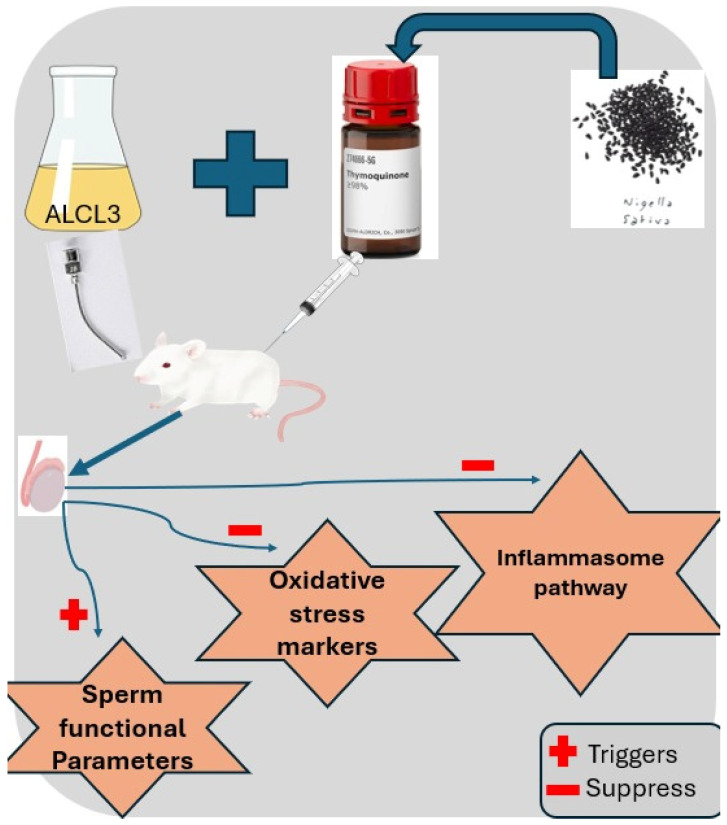
Schematic illustration showing that AlCl_3_ induces oxidative stress and inflammasome activation, leading to reduced sperm functional parameters, while TQ counteracts these effects, suppressing oxidative and inflammatory pathways and improving sperm quality and function. (+) denotes activation; (−) denotes suppression.

Thymoquinone attenuated AlCl_3_-induced testicular injury, restored rat and testicular weights, sperm quality, sex hormone levels, testicular enzymes, and antioxidant markers, and histological testicular structure. This may occur through the suppression of testicular NLRP3/caspase 1/IL-1β inflammasome signaling and the polarization of macrophages toward an M2 anti-inflammatory phenotype. Figure 10 shows a graphical abstract for the research study.

## 6. Study Limitations

This study reported the promising action of TQ in suppressing AlCl_3_-induced testicular injury by inhibiting the NLRP3/caspase 1/IL-1β inflammasome pathway and polarizing macrophages toward an M2 anti-inflammatory phenotype. However, several limitations remain. We studied only one dose of AlCl_3_ and TQ; therefore, we recommend investigating different doses to ensure dose dependence and determine the optimal dose. We recommend adding an untreated control group. In addition, we recommend conducting a longer experiment to cover the entire spermatogenic cycle. We suggest that future studies could distinguish between pro- and mature IL-1β to confirm this activation further. Moreover, the NLRP3/caspase 1/IL-1β inflammasome was the primary focus of this investigation; however, IL-18 is another key cytokine activated by the NLRP3 inflammasome through caspase-1–mediated cleavage, and should be studied in parallel with IL-1β. In the present work, we focused on the detection of NLRP3, caspase-1, and IL-1β as representative markers of inflammasome activation, using immunohistochemistry. However, Western blotting, gene expression studies, immunofluorescence co-localization, and systemic cytokine profiling (including serum IL-1β) are generally required to validate and quantify protein expression levels and to provide a more comprehensive assessment of inflammasome activation and its systemic consequences. Finally, we suggest that future studies include the evaluation of functional endpoints, such as epididymal sperm DNA integrity and fertilization potential (using IVF assays or mating trials).

## Data Availability

The original contributions presented in this study are included in the article/Supplementary Material. Further inquiries can be directed to the corresponding author(s).

## Supplementary Materials

The following supporting information can be downloaded at: https://www.mdpi.com/article/10.3390/cells14231906/s1, Figure S1: Epididymal sperm smear; Table S1: (Body and testicular weights); Table S2: semen analysis; Table S3: Hormonal assay; Table S4: Testicular enzymes; Table S5: Oxidative markers; Table S6: Immunohistochemical study.
