# Supplementary material for: Thymoquinone Attenuates Aluminum Chloride-Induced Testicular Injury by Inhibiting NLRP3/Caspase 1/IL-1β Inflammasome Signaling and Polarizing the Macrophages Toward Anti-Inflammatory M2 Phenotype
Source: Cells. 2025 Dec 1;14(23):1906. doi: 10.3390/cells14231906 (PMC12691080; doi:10.3390/cells14231906)
Supplement: Supplementary file 1 [file cells-14-01906-s001.zip › cells-4001713-supplementary.pdf]

Supplementary table S1 (Body and testicular weights)

parameter	Groups				F value	P value
	Negative control [n=6]	TQ control [n=6]	AlCl3 [n=6]	AlCl3 + TQ [n=6]		
Body Weight in gm	257.483±1.91 A	257.91±1.8 A	216.315±0.856 B	240.481±1.275 C	165.508	**<0.0005**
Testicular Weight in gm	2.00±0.009 A	1.99±0.009 A	1.48±0.029 B	1.685±0.021 C	166.421	**<0.0005**

Data are tabulated as mean ± standard error. ANOVA followed by the LSD test was performed, assuming equal variances for body weights. For testicular weight, the Games-Howell test was used when equal variance was not assumed. P values were mentioned as letters, where different letters = significant difference

Supplementary table S2: semen analysis

parameter	Groups				F value	P value
	Negative control [n=6]	TQ control [n=6]	AlCl3 [n=6]	AlCl3 + TQ [n=6]		
Sperm Count 10^6/ml	39.093±1.29 A	39.86±1.20 A	191.123±0.831 B	32.95±0.77 C	80.57	**<0.0005**
Sperm Motility %	83.02±0.79 A	84.96±0.79 A	46.181±0.72 B	67.585±1.49 C	321.313	**<0.0005**
Abnormal Sperm Morphology %	12.0433±0.63 A	10.628±0.357 A	38.916±0.66 B	18.521±0.608 C	507.664	**<0.0005**

Data are tabulated as mean ± standard error. ANOVA followed by the LSD test was done assuming equal variances. P values were mentioned as letters, where different letters = significant difference

Supplementary Table S3: hormonal assay

parameter	Groups				F value	P value
	**Negative control [n=6]**	**TQ control [n=6]**	**AlCl3 [n=6]**	**AlCl3 + TQ [n=6]**		
FSH mIU/ml	3.43±0.03 A	3.42±0.031 A	2.38±0.07 B	3.16±0.09 C	61.605	**<0.0005**
LH mIU/ml	2.97±0.06 A	2.96±0.07 A	1.79±0.08 B	2.57±0.1 C	45.118	**<0.0005**
Testosterone ng/ml	3.98±0.05 A	3.99±0.05 A	2.08±0.18 B	3.47±0.112 C	60.928	**<0.0005**

Data are tabulated as mean ± standard error. ANOVA followed by the LSD test was done assuming equal variances for LH, while for FSH and testosterone, the Games-Howell test was used as equal variance was not assumed. P values were mentioned as letters, where different letters = significant difference

Supplementary Table S4: Testicular enzymes:

parameter	Groups				F value	P value
	**Negative control [n=6]**	**TQ control [n=6]**	**AlCl3 [n=6]**	**AlCl3 + TQ [n=6]**		
ACP level in U·g prot^−1	157.23±3.42 A	158.16±2.72 A	217.76±4.483 B	143.671±0.515 C	110.469	**<0.0005**
SDH level in U·mg prot^−1	11.11±0.13 A	11.06±0.11 A	8.803±0.141 B	10.241±0.062 C	84.899	**<0.0005**
LDH level in U·g prot ^−1	1123.41±14.39 A	1126±18.19 A	1288.60±15.28 B	1049.94±7.43 C	49.091	**<0.0005**
ACP/LDH Ratio	0.14±0.003 A	0.14±0.003 A	0.16±0.004 B	0.13±0.001 C	26.281	**<0.0005**

Data are tabulated as mean ± standard error. ANOVA followed by the LSD test was done assuming equal variances, except for LDH, where the Games-Howell test was used as equal variance was not assumed. P values were mentioned as letters, where different letters = significant difference

Supplementary Table S5: oxidative markers

parameter	Groups				F value	P value
	**Negative control [n=6]**	**TQ control [n=6]**	**AlCl3 [n=6]**	**AlCl3 + TQ [n=6]**		
NO (µmol/g tissue)	16.061±0.475 A	16.701±0.382 A	40.576±0.46 B	24.698±0.42 C	684.381	**<0.0005**
MDA (nmol/g tissue)	349.473±2.48 A	339.33±4.47 A	793.655±8.94 B	497.883±3.31 C	1532.202	**<0.0005**
SOD (u/g tissue)	48.973±0.64 A	47.078±0.27 A	33.625±0.58 B	47.088±0.64 C	160.935	**<0.0005**
CAT (u/g tissue)	73.978±0.72 A	72.685±0.40 A	48.628±0.32 B	69.42±0.36 C	606.952	**<0.0005**

Data are tabulated as mean ± standard error. ANOVA followed by the LSD test was done assuming equal variances, except for MDA & CAT, where the Games-Howell test was used as equal variance was not assumed. P values were mentioned as letters, where different letters = significant difference

Supplementary Table S6: immune histochemical expression

parameter	Groups				F value	P value
	**Negative control [n=6]**	**TQ control [n=6]**	**AlCl3 [n=6]**	**AlCl3 + TQ [n=6]**		
Mean Caspase 1 Area Percentage%	6.94±0.59 A	6.97±0.54 A	51.07±0.789 B	20.08±1.34 C	561.624	**<0.0005**
Mean IL1b Area Percentage%	5.43±0.14 A	5.80±0.33 A	41.21±1.23 B	12.55±0.35 C	647.413	**<0.0005**
Mean NLRP3 Area Percentage%	4.61±0.37 A	4.41±0.36 A	56.84±1.64 B	28.14±1.56 C	457.403	**<0.0005**
CD68+ Area Percentage	4.001±0.42 A	3.97±0.38 A	17.07±0.504 B	8.314±0.279 C	228.829	**<0.0005**
CD163+ Area Percentage	4.415±0.152 A	4.591±0.156 A	3.783±0.231 B	8.532±0.226 C	122.877	**<0.0005**
CD163+/CD68+ Ratio	1.171±0.133 A	1.208±0.123 A	0.222±0.014 B	1.035±0.056 C	23.507	**<0.0005**

Data are tabulated as mean ± standard error. ANOVA followed by Games-Howell test as equal variance was not assumed. P values were mentioned as letters, where different letters = significant difference

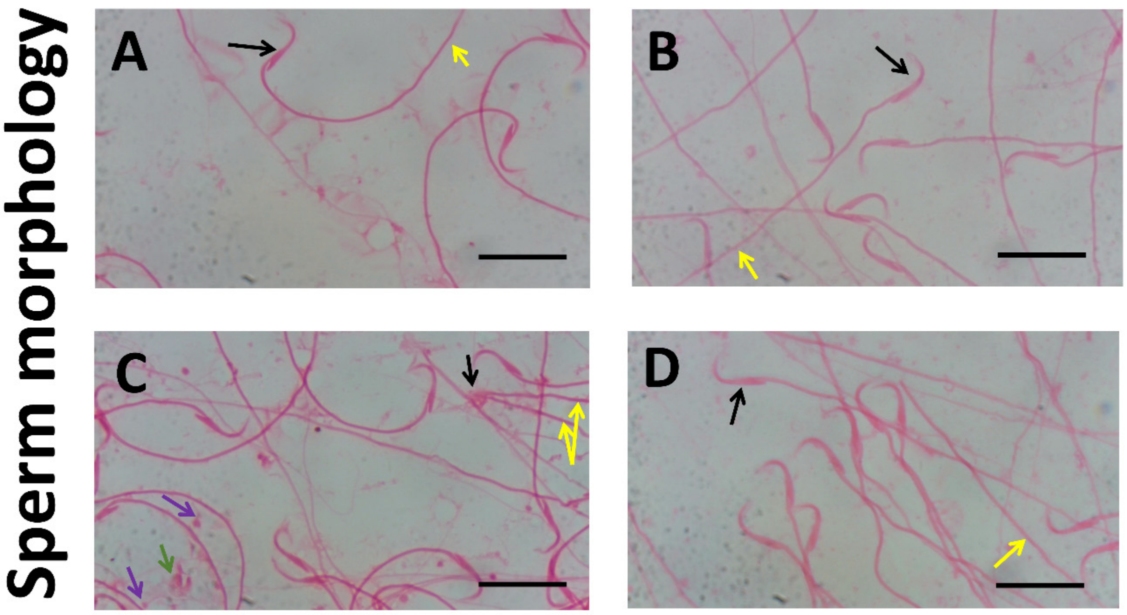

Supplementary Fig. 1: (A-D) Epididymal sperm smear stained by eosin (×1000) of the negative control, TQ, AlCl3, and AlCl3 + TQ groups (A, B, C, and D; respectively). H&E of the negative control and TQ groups showed normal sperm morphology, normal shape of head (black arrows), and tails (Yellow arrows). The AlCl3 group showed a degenerated head (black arrow), detached head (green arrow), double tail (yellow arrows), and dwarf sperms (purple arrows). The AlCl3 + TQ group showed relative restoration of the normal sperm morphology, normal shape of head (black arrows), and tails (Yellow arrows).
